# COVID-19-Related Fear, Risk Perception, and Safety Behavior in Individuals with Diabetes

**DOI:** 10.3390/healthcare9040480

**Published:** 2021-04-18

**Authors:** Venja Musche, Hannah Kohler, Alexander Bäuerle, Adam Schweda, Benjamin Weismüller, Madeleine Fink, Theresa Schadendorf, Anita Robitzsch, Nora Dörrie, Susanne Tan, Martin Teufel, Eva-Maria Skoda

**Affiliations:** 1 Clinic for Psychosomatic Medicine and Psychotherapy LVR University Hospital, University of Duisburg-Essen, 45147 Essen, Germany; alexander.baeuerle@uni-due.de (A.B.); adam.schweda@lvr.de (A.S.); BenjaminMaurice.Weismueller@lvr.de (B.W.); madeleine.fink@uni-due.de (M.F.); Theresa.Schadendorf@lvr.de (T.S.); Anita.Robitzsch@uni-due.de (A.R.); nora.doerrie@uni-due.de (N.D.); martin.teufel@uni-due.de (M.T.); Eva-maria.Skoda@uni-due.de (E.-M.S.); 2 Department of Endocrinology, Diabetes and Metabolism, University of Duisburg-Essen, University Hospital Essen, 45147 Essen, Germany; susanne.tan@uk-essen.de

**Keywords:** diabetes, COVID-19, COVID-19-related fear, safety behavior, risk perception

## Abstract

(1) The aim of the study is to assess the psychological burden of individuals with diabetes during the COVID-19 pandemic in comparison to matched controls. (2) Over the course of eight weeks, 9 April to 3 June 2020, 253 individuals with diabetes and 253 matched controls, using Propensity Score Matching (PSM), participated in this cross-sectional study. Participants completed an anonymous survey including demographics, depressive symptoms (PHQ-2), generalized anxiety (GAD-7), COVID-19-related fear, risk perception, and safety behavior. (3) While patients with diabetes expected their risk of infection similar to controls, they reported a higher probability of the occurrence of symptoms, severe course, and dying of COVID-19. Patients with diabetes showed no elevated generalized anxiety or depressive symptoms. However, they reported higher COVID-19-related fear and more adherent and dysfunctional safety behavior compared to controls. (4) From a public health view, it seems encouraging that despite the somatic risk condition, generalized anxiety and depression are not higher in patients with diabetes than in controls. Patients with diabetes report higher COVID-19-related fear, increased risk perception, and behavioral changes. This suggests that individuals with diabetes, as a significant risk group of severe COVID-19, show an adequate perception and functional reaction to the current pandemic.

## 1. Introduction

The current pandemic caused by the novel coronavirus SARS-CoV-2 (severe acute respiratory syndrome CoV-2, shortened as coronavirus) has been a constant challenge for politics, the health system, research, and everyday life. Due to the increasing number of infections, it is still a highly topical issue. With an acute infection rate of 108,153,741confirmed cases worldwide and 2,381,295 deaths (until 14 February 2021), this is one of the largest pandemics in history [1]. Analyses of fatalities and patients who have had a severe course of COVID-19 (coronavirus disease-19) show that people with the number of chronic diseases are at increased risk [2]. Further medical research has shown that individuals with diabetes, in particular, are at increased risk for severe COVID-19 [3].

Individuals with diabetes form a special group with significant risk due to their underlying disease and the frequent occurrence of comorbidity. In the literature, there is the hypothesis that patients with diabetes are vulnerable to an inflammatory storm [4]. Such an acute release of cytokines can induce a rapid progression of COVID-19. Another hypothesis is that the coronavirus has an impact on glucose metabolism, thereby altering blood sugar levels and the amount of medication required [4]. It has been further shown that medications that reduce the intensity of COVID-19 symptoms can influence the blood sugar level and the efficiency of diabetes medication [5].

With regard to the current research situation, there is a rapidly growing body of new literature on COVID-19, but little is known about the psychological burden on people with diabetes during the pandemic. In previous studies, it has been shown that the psychological burden on people during the COVID-19 pandemic is increasing rapidly. The population worldwide suffers from the uncertainty of the pandemic. An increase in depression, anxiety, sleeping problems, and stress has been reported [6,7,8,9,10]. Not only on the psychological burden on people increasing in the current situation, but the COVID-19 pandemic may also have a long-term effect on the population [11,12]. According to the literature, people who belong to a risk group on account of their chronic diseases report higher levels of worries and fears due to COVID-19 and show overall an increased psychological burden [6,7,9].

Individuals with diabetes are a highly vulnerable group of patients. Both depression and anxiety disorders are diagnosed more frequently in patients with diabetes than in people without. Studies have shown that the risk of developing depression is 24% higher in patients with type 2 diabetes (T2DM) than in individuals without diabetes [13]. The prevalence of developing depression is also significantly higher among patients with T2DM compared to individuals without diabetes (17.6% vs. 9.8% odds ratio = 1.6, 95% confidence interval 1.2–2.0) [14]. The prevalence of anxiety disorders is higher in people with diabetes than in the general population. According to the literature, 14 and 27% of diabetics suffer from a generalized and sub-syndromal anxiety disorder, respectively. A total of 40% have elevated anxiety symptoms compared to persons without diabetes [15].

With the knowledge of the vulnerability of these individuals and the current situation, we conducted a study to investigate the psychological burden of the COVID-19 pandemic on patients with diabetes. The aim of this study was to shed light on the psychological burden and the needs of individuals with diabetes during the pandemic. The topic of investigation was generalized anxiety, depressive symptoms, COVID-19-related fear, risk perception, and associated behavioral changes (i.e., adherent and dysfunctional safety behavior) in patients with diabetes in comparison to a matched control group. We hypothesized diagnosis of diabetes as a risk factor for severe COVID-19 to be associated with generalized anxiety, depressive symptoms, and COVID-19-related fear. Furthermore, we expected that individuals with diabetes report more behavioral changes, i.e., increased safety behavior, compared to controls. Both groups were expected to indicate similar subjective risk perception of infection with COVID-19. As a risk group, patients with diabetes were expected to report higher (subjective) chances of the occurrence of symptoms, a severe course, and dying of COVID-19.

## 2. Materials and Methods

### 2.1. Participants and Procedure

Over the course of eight weeks, 9 April to 3 June 2020, 253 patients with diabetes and 253 matched controls participated in this cross-sectional study. The participants were recruited via online channels (e.g., online newspaper), social media (e.g., Facebook), and print media. The control group was sampled from a nationwide study in Germany reported in previous research [6]. The eligibility requirement was adult age (≥18 years), a good command of the German language and Internet access for all participants, and a diagnosis of diabetes for the patient sample. All participants provided written informed consent to take part in the anonymous online survey. The study was approved by the Ethics Committees of the University Hospital Essen (20-9307-BO).

### 2.2. Propensity Score Matching

Patients with diabetes are, most likely, not very representative of the whole population, and thus, not directly comparable to a convenience sample. In order to make these groups comparable, we applied propensity score matching (PSM), a technique that is able to create adequate control groups based on a number of previously differing covariates, such as age, education, and sex. By maximizing the similarity between the reference and the control group in a number of potentially confounding variables, it reduces the risk of obtaining results that are actually accountable to one or more secondary attributes of the reference group instead of the primary selection criterion, as in our case, diabetes diagnosis. To do so, the R package MatchIt [16] was used to apply the nearest neighbor matching method. The control group was matched based on the variables education, age, marital status, sex, community size, and the presence of mental disorders. A criterion of 0.1 is applied for considering the standardized difference between groups negligible [17]. None of the comparisons exceeded this criterion.

### 2.3. Assessment Instruments

The online survey contained items on socio-demographic and medical details, validated assessment instruments, and self-generated items on COVID-19-specific aspects.

Socio-demographic and medical details. Socio-demographic data were assessed, including sex, age, marital status, educational level, and city size. In addition, patients with diabetes were asked about their diagnosed type of diabetes, time since diagnosis, how well the disease is controlled, and whether there are concomitant diseases.

Generalized Anxiety Disorder Scale-7 (GAD-7): The GAD-7 consists of seven items assessing self-reported anxiety and its severity over the past 2 weeks. Answers are given on a 4-point Likert scale, ranging from 0 = not at all to 3 = nearly every day. Possible total scores range from 0 to 21, with scores of 5, 10, and 15 serving as thresholds for mild, moderate, and severe anxiety, respectively. The GAD-7 showed high reliability and validity in health care and research [18]. In the present sample, the internal consistency was high with a Cronbach’s α of 0.90.

Patient Health Questionnaire-2 (PHQ-2): The PHQ-2 consists of two items assessing depressive symptoms over the past 2 weeks. Answers are given on a 4-point Likert scale, ranging from 0 = not at all to 3 = nearly every day. Possible total scores range from 0 to 6, with scores of 3 serving as a cut-off for major depression symptoms [19]. In the present sample, the internal consistency was high with a Cronbach’s α of 0.84.

Subjective level of information about COVID-19: The subjective level of information about COVID-19 (e.g., I feel informed about measures to avoid infection with COVID-19) was assessed by three items. Answers were given on a 7-point Likert scale ranging from 1 = strongly disagree to 7 = strongly agree. Its internal consistency was good with a Cronbach’s α of 0.79 [6].

COVID-19-related fear: COVID-19-related fear was assessed by a single item (I worry about COVID-19). Answers were given on a 7-point Likert scale ranging from 1 = strongly disagree to 7 = strongly agree. Thus, a higher score indicates a higher level of COVID-19-related fear [6,20,21,22].

Adherent Safety Behavior (ASB) and Dysfunctional Safety Behavior (DSB): Safety behavior was assessed by the 4-item sub-scale of ASB with a Cronbach’s alpha of 0.83 and the 4-item sub-scale of DSB with a Cronbach’s alpha of 0.77. Answers were assessed on a 7-point Likert scale ranging from 1 = strongly disagree to 7 = strongly agree [20].

Subjective risk perception: The subjective risk perception of the infection with COVID-19, suffering from COVID-19-related symptoms, having a severe course, and dying of COVID-19 was recorded in percent (0–100%).

### 2.4. Statistical Analyses

Matching was completed with the R package MatchIt [23]. Further statistical analyses were performed using SPSS Statistics version 26 (IBM, NY, USA). Considering the present sample size, a normal distribution of the variables was assumed [24]. Independent sample t-tests were calculated to investigate differences between individuals with diabetes and healthy controls in generalized anxiety, depressive symptoms, COVID-19-related fear, subjective risk perception, subjective level of information about COVID-19, and associated behavioral changes (i.e., ASB and DSB). The level of significance was set at α = 0.05 (two-sided tests).

## 3. Results

### 3.1. Participant Characteristics

The participants were 253 individuals with diabetes and 253 healthy controls, who were matched to those with diabetes based on their demographic data. Most of the patients with diabetes were female (74.3%), aged between 35 and 64 years (72.0%), and married (46.2%). Most patients suffered from type 1 diabetes (66.8%), with a mean diabetes duration of 17.24 years (SD = 0.87). Most of the controls were female (77.5%), aged between 35 and 64 years (70.8%), and married (47.0%). For all details, see Table 1.

### 3.2. Generalized Anxiety and Depressive Symptoms

The results of the independent sample *t*-tests revealed no significant differences between patients with diabetes and healthy controls concerning generalized anxiety, *t*(504) = −0.800, *p* = 0.424, and depressive symptoms, *t*(504) = −0.389, *p* = 0.697. For all details, see Table 2. A total of 21.3% of the individuals with diabetes and 19.3% of the controls reported scores above the cut-off, indicating depressive symptoms. Patients and controls reported similar anxiety scores. A total of 29.2% of the patients (vs. 36.8%) reported mild symptoms, 16.2% (vs. 15.4%) moderate symptoms, and 7.9% (vs. 7.1%) severe generalized anxiety symptoms compared to the controls (see Table 2).

### 3.3. COVID-19-Related Fear, Subjective Level of Information, and Safety Behavior

Patients with diabetes reported significantly higher COVID-19-related fear compared to controls, *t*(504) = 2.780, *p* = 0.006, *d* = 0.246. Patients and controls felt equally well informed about COVID-19, *t*(504) = 1.647, *p* = 0.100. In regard to safety behavior, patients with diabetes reported higher levels of ASB, *t*(504) = 2.757, *p* = 0.006, *d* = 0.239, and DSB compared to healthy controls, *t*(504) = 2.084, *p* = 0.038, *d* = 0.180. For an overview, see Table 3.

### 3.4. Subjective Risk Perception

**Figure 1:**
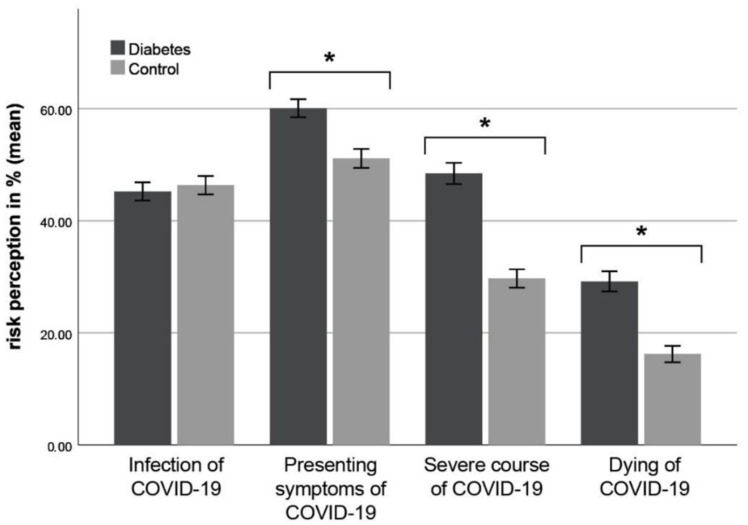
Risk perception in patients with diabetes compared to controls. * *p* < 0.001, error bars: 95%.

Independent sample t-tests revealed no significant differences between patients with diabetes and controls concerning the subjective risk perception of infection with COVID-19, *t*(504) = −0.478, *p* = 0.633. However, patients with diabetes reported a significantly higher probability of the occurrence of symptoms, *t*(504) = 3.842, *p* < 0.001, *d* = 0.342, a severe course, *t*(504) = 7.524, *p* < 0.001, *d* = 0.669 and dying of COVID-19, *t*(504) = 5.583, *p* < 0.001, *d* = 0.497. For an overview, see Figure 1.

## 4. Discussion

The present study examined the psychological burden of individuals with diabetes during the COVID-19 pandemic. In addition, to the best of our knowledge, this study is to date the first to investigate the psychological burden of individuals with diabetes in comparison to matched controls from a large control sample. Compared to the controls, individuals with diabetes did not show elevated generalized anxiety or depressive symptoms. They rated their probability of infection of COVID-19 similarly to controls. However, individuals with diabetes reported a significantly higher probability of symptoms occurrence, severe course, and death from COVID-19. Furthermore, they reported increased COVID-19-related fear and associated behavioral changes, i.e., more ASB, such as increased hand hygiene, and DSB, such as buying larger quantities of staple food, compared to the controls.

To recapitulate briefly, diabetes diagnosis strains the mental health of affected individuals [13,14,15]. It was therefore expected that individuals with diabetes would be particularly threatened by the COVID-19 pandemic. However, the present data show that there is no increased generalized anxiety or depressive symptoms in individuals with diabetes compared to controls, suggesting that the current pandemic does not cause an increased generalized psychological burden, specifically in individuals with diabetes. During the survey period, different protection measures were implemented in Germany. The beginning of the survey was during the lockdown phase, including the closure of public facilities and contact bans. At the end of April, it also became mandatory to wear face masks in public places [21]. During this time, there was a stepwise easing of the measures. However, normality was far from being achieved. This has to be considered as a possible additional burden and confounder besides the fear about a possible infection/illness of COVID-19. There is a growing body of literature that shows that psychological distress is increased during the ongoing pandemic. In line with previous research [6], both groups report an increased prevalence of depressive symptoms and generalized anxiety compared to previously published studies in Germany [18,25]. Compared to studies conducted before the pandemic, the measured score for depressive symptoms was significantly higher in both groups. Before the pandemic, only 5.6% of the population had an increased prevalence of depressive symptoms [6].

While generalized anxiety and depressive symptoms did not differ between the groups, individuals with diabetes reported increased COVID-19-related fear compared to controls. In the context of the ongoing pandemic, this could be interpreted as a functional emotional response and might lead to the need for security, which is reflected in increased safety behavior (i.e., ASB and DSB). The increased fear could also be seen as a warning function and could be used as a behavioral or adherence preparation. The more recent literature suggests a distinction between the fear of COVID-19 and generalized anxiety [26,27,28]. Fear is, as the word already implies, the worry of a situation or object. This can be expressed in purposeful activism. In contrast, there is generalized anxiety, which is often perceived as panic. It can possibly limit the senses and manifest itself in avoidance behavior.

The elevated COVID-19-related fear could be a consequence of the increased risk perception reported by diabetes patients. While diabetes patients rate their probability of infection similar to controls, they suspect an increased risk of developing symptoms, a severe course, and dying of COVID-19 compared to the control group. These results are not surprising since individuals with diabetes who identified early on as a risk group for severe COVID-19 might indeed have an increased likelihood of developing symptoms, a severe course, and dying from COVID-19 [29]. Thus, the increased risk perception could, to a certain extent, represent a realistic assessment of their objective risks in the event of infection. However, these risk assessments are overestimated compared to the current data.

In general, the manifestation index of COVID-19 is estimated to be 55–85%. This is an indication of how many infected people ultimately developed symptoms of the disease. In Germany, 3.8% of people with a confirmed COVID-19 infection died as a result of the disease [30]. In addition, it is assumed that 81% of the people who were diagnosed with COVID-19 had a mild course of the disease, about 14% a more severe course, and about 5% a critical course [31]. A recent study has shown that COVID-19 patients diagnosed with pre-existing diabetes have a twofold increased risk of a severe course of the disease (*n* = 22 studies; random-effects odds ratio 2.10, 95% CI 1.71–2.57; I2 = 41.5%) and about a threefold increased risk of dying from COVID-19 (*n* = 15 studies; random-effects odds ratio 2.68, 95% CI 2.09–3.44; I2 = 46.7%) [32]. A report from China estimated that 49% of the patients with a critical severe course of COVID-19 eventually died. The case-fatality rate (CFR, patients with a proven infection relative to death rates from COVID-19) was increased among individuals with pre-existing risk diseases such as diabetes (CFR increased about 7.3%) [31].

An increased risk might be associated with poorly controlled diabetes, diabetes-related comorbidities, use of immunosuppressive medication due to organ transplantation, advanced age, as well as pre-existing diseases [33]. In our study, it could be shown that individuals with diabetes estimated their risk of developing symptoms with a mean of 60.08% (non-diabetic respondents with a mean of 51.12%), their risk of a severe course with a mean of 48.46% (respondents without diabetes with a mean of 29.69%) and the risk of dying of COVID-19 with a mean of 29.18% (non-diabetic respondents with a mean of 16.22%). Compared to the current data from daily clinical routine, both groups overestimated their risks. In this context, it is important to mention that the diabetes control status varied among the patient sample. This could have important implications in regard to the perceived risk and health-related safety behavior in diabetes patients.

Besides the emotional consequences that are reflected in increased COVID-19-related fear, diabetics also indicated generally more safety behavior, i.e., increased ASB, as well as increased DSB. This could be interpreted as a functional behavioral response as a result of their estimation as a risk group with increased risk perception and increased COVID-19-related fear. This is consistent with previous research. In cross-sectional studies, it has been shown that health-related safety behavior, including increased hand hygiene, could be significantly predicted by fear of the current pandemic [21] and could be interpreted as a functional behavioral response in the context of the COVID-19 pandemic [27].

With the knowledge of the needs and concerns of individuals with diabetes, it is important to implement care structures. These services should be low-threshold and freely available so that they are easily accessible to everyone. There are already various offers for mentally burdened people during the COVID-19 pandemic in Germany. These services include telephone consultations as well as online support regarding, e.g., mindfulness and cognitive-behavioral stress reduction techniques [34]. Experiences from previous pandemics have shown that people are still mentally stressed months after a pandemic [35]. The services currently available are aimed primarily at the general population. There is currently no specific support for individuals with diabetes.

### Limitations

The strength of the present study is that it examines the psychological burden of individuals with diabetes during the COVID-19 pandemic in comparison to PSM-based matched controls. However, some limitations must be taken into account. Due to the cross-sectional design, no statements can be made about causality. The study results are based on self-reports. Thus, objective verification of the diabetes diagnosis is not possible. The possibility of selection biases exists. The generalizability of the study results could be questioned as the majority of patients with diabetes report a diagnosis of type 1 diabetes. This does not reflect the actual distribution of type 1 and 2 diabetes in society. There are significantly more individuals diagnosed with type 2 diabetics. Therefore, this limitation should be considered in the interpretation of the results. No validated assessment tool for the COVID-19-related fear was available at the time the study was conducted. Using one single item, the validity of the COVID-19-related fear assessment is in question. Unfortunately, a possible validated assessment instrument was available just after the survey was conducted [26].

## 5. Conclusions

In conclusion, the current COVID-19 pandemic has a significant impact on the population and especially on people with diabetes and on their daily lives, and is a threatening situation overall. Individuals with diabetes show levels of generalized anxiety and depressive symptoms are comparable to those of healthy controls. However, individuals with diabetes report more COVID-19-related fear, increased risk perception, and behavioral changes, i.e., increased safety behavior. This suggests that individuals with diabetes, as a significant risk group for a potential severe COVID-19 course, show an adequate perception and functional reaction to the current pandemic. Nevertheless, individual support systems should be implemented to address the concerns of these groups and provide long-term mental health support.

## Figures and Tables

**Table 1:** Socio-demographic and medical characteristics.

Characteristics	Diabetes Patients		Healthy Controls	
	*N*	%	*N*	%
**Sex**				
Female	188	74.3	196	77.5
Male	65	25.7	57	22.5
**Age**				
18–34 years	62	24.5	68	26.8
35–54 years	131	51.8	136	53.8
55–74 years	59	23.4	47	18.6
>74 years	1	0.4	2	0.8
**Marital status**				
Single	64	25.3	66	26.1
Married	117	46.2	119	47.0
In a relationship	55	21.7	50	19.8
Divorced/separated	14	5.5	15	5.9
Widowed	3	1.2	3	1.2
**Educational level**				
University education	72	28.5	71	28.1
Higher education entrance qualification	87	34.4	84	33.2
Intermediate secondary education	63	24.9	57	22.5
Lower secondary education	29	11.5	38	15.0
No qualification	2	0.8	3	1.2
**City size**				
100,000 residents	84	33.2	89	35.2
20,000 residents	72	28.5	68	26.9
5000 residents	41	16.2	37	14.6
<5000 residents	56	22.1	59	23.3
**Diabetes mellitus diagnosis**				
Type 1 diabetes	169	66.8	-	-
Type 2 diabetes	74	29.2	-	-
Other diabetes diagnosis	10	4.0	-	-
**Assessment of diabetes control**				
Good	126	49.8	-	-
Average	107	42.3	-	-
Not good	14	5.5	-	-
I can’t tell	6	2.4	-	-
**Accompanying illness(es)**				
None	122	48.2	-	-
One	54	21.3	-	-
Two	30	11.9	-	-
More than two	47	18.6	-	-
**Mental disorder(s)**				
No	184	72.7		
Yes	69	27.3		
**Total**	253	100.0	253	100.0

**Table 2:** Comparisons between patients with diabetes and healthy controls.

Assessment instruments	Diabetes Patients (*n* = 253)	Healthy Controls (*n* = 253)	Statistical Analyses		
	*M* (*SD*)	*M* (*SD*)	*t*	*p*	*d*
GAD-7	6.09 (5.20)	6.45 (5.02)	−0.800	0.424	-
PHQ-2	1.50 (1.75)	1.56 (1.67)	−0.389	0.697	-
COVID-19-related fear	4.81 (1.69)	4.35 (2.04)	2.780	0.006	0.246
Subjective level ofinformation	5.83 (0.94)	5.67 (1.19)	1.647	0.100	-
ASB	5.86 (1.15)	5.52 (1.65)	2.757	0.006	0.239
DSB	2.92 (1.35)	2.68 (1.31)	2.084	0.038	0.180

*Note*. Mean parameter values for each of the analyses are shown for patients with diabetes (*n* = 253) and healthy controls (*n* = 253), as well as the t-tests (assuming unequal variance). Significant at the 0.05 level. GAD-7 = Generalized Anxiety Disorder Scale-7. PHQ-2 = Patient Health Questionnaire-2. ASB = adherent safety behavior, DSB = dysfunctional safety behavior.

**Table 3:** Prevalence of generalized anxiety symptoms and depressive symptoms stratified by diabetes diagnosis.

Diabetes Mellitus Diagnosis					Healthy Controls
	Type I (*n* = 169)	Type II (*n* = 74)	Other (*n* = 10)	(*n* = 253)	(*n* = 253)
GAD-7					
<5	78 (46.2%)	34 (45.9%)	6 (60.0%)	118 (46.6%)	103 (40.7%)
≥5	52 (30.8%)	20 (27.0%)	2 (20.0%)	74 (29.2%)	93 (36.8%)
≥10	29 (17.2%)	11 (14.9%)	1 (10.0%)	41 (16.2%)	39 (15.4%)
≥15	10 (5.9%)	9 (12.2%)	1 (10.0%)	20 (7.9%)	18 (7.1%)
PHQ-2					
<3	136 (80.5%)	55 (74.3%)	8 (80.0%)	199 (78.7%)	204 (80.6%)
≥3	33 (19.5%)	19 (25.7%)	2 (20.0%)	54 (21.3%)	49 (19.4%)

Note. GAD-7 = Generalized Anxiety Disorder Scale-7, sum scores of ≥5, ≥10, and ≥15 indicate mild, moderate, and severe generalized anxiety symptoms, respectively. PHQ-2 = Patient Health Questionnaire-2, sum scores of ≥3 indicate major depression symptoms. Others include all patients who are not classified as diabetes type 1 or 2.

## Data Availability

The raw data supporting the conclusions of this article will be made available by the authors on request.
